# Supplementary material for: Temporal trends in overweight and obesity and chronic disease risks among adolescents and young adults: A ten-year review at a tertiary institution in Nigeria
Source: PLoS One. 2023 Apr 5;18(4):e0283210. doi: 10.1371/journal.pone.0283210 (PMC10075485; doi:10.1371/journal.pone.0283210)
Supplement: Attachment — Submitted filename: Response to Reviewers.docx [file pone.0283210.s001.docx]

**Rebuttal letter: response to reviewers**

PONE-D-22-08089

**Temporal Trends in Obesity and Chronic disease risks Among Adolescents and Young Adults: A Ten-year Review at a Tertiary Institution in Nigeria**

PLOS ONE

| **Journal Requirements:** |  |
| --- | --- |
| 1. Please ensure that your manuscript meets PLOS ONE's style requirements, including those for file naming. The PLOS ONE style templates can be found at   https://journals.plos.org/plosone/s/file?id=wjVg/PLOSOne_formatting_sample_main_body.pdf and  https://journals.plos.org/plosone/s/file?id=ba62/PLOSOne_formatting_sample_title_authors_affiliations.pdf | Thank you for the guidance, the PLOS ONE’s style requirements have been incorporated accordingly in all sections. |
| 2. In the ethics statement in the Methods and online submission information, please ensure that you have specified (1) whether consent was informed and (2) what type you obtained (for instance, written or verbal, and if verbal, how it was documented and witnessed). If your study included minors, state whether you obtained consent from parents or guardians. If the need for consent was waived by the ethics committee, please include this information.   If you are reporting a retrospective study of medical records or archived samples, please ensure that you have discussed whether all data were fully anonymized before you accessed them and/or whether the IRB or ethics committee waived the requirement for informed consent. If patients provided informed written consent to have data from their medical records used in research, please include this information. | This is a retrospective study of medical records. All data were fully anonymized before we accessed them. The ethical approval for this study was provided by the Social Sciences and Humanities Research Ethics Committee (SSHREC), University of Ibadan (UI), Nigeria [UI/SSHEC/2020/0021]. The SSHREC waved the need for informed consent for the study. Line 204-211.  This information has been included in the ethics statement. Thank you. |
| 3. Thank you for stating the following in the Acknowledgments Section of your manuscript:   “A special appreciation goes to the 12th Vice Chancellor of the University of Ibadan, Prof. Abel Idowu Olayinka, who provided funding and institutional support to AOO for capacity building and the conduct of the study.”  We note that you have provided additional information within the Acknowledgements Section that is not currently declared in your Funding Statement. Please note that funding information should not appear in the Acknowledgments section or other areas of your manuscript. We will only publish funding information present in the Funding Statement section of the online submission form.  Please remove any funding-related text from the manuscript and let us know how you would like to update your Funding Statement. Currently, your Funding Statement reads as follows:  “The authors received no specific funding for this work.”  Please include your amended statements within your cover letter; we will change the online submission form on your behalf. | Including funding information in our acknowledgement is regretted. We have deleted the funding information in the Acknowledgement Section. This section has been updated and should read:  “AOO is grateful to The Royal Tropical Institute (KIT), Amsterdam, the Netherlands, through Orange knowledge Scholarship, which provided training on the ‘Control Strategies for Communicable and Non-communicable Diseases.’ Also, the Bernard Lown Scholars Program, Harvard T. Chan School of Public Health, provided Fellowship training in Cardiovascular Disease Prevention and support to AOO for the conduct of this study. A special appreciation goes to the 12th Vice-Chancellor of the University of Ibadan, Prof. Abel Idowu Olayinka, who provided institutional support to AOO for capacity building and the conduct of the study”.  Amendments to the Acknowledgement section and Funding statement have been included in the cover letter for your kind attention.  In the online submission form we would like to update the funding statement to read:  AOO received financial support for capacity building and the conduct of this study from the office of the 12^th^ Vice-Chancellor, University of Ibadan, Nigeria. None of the co-authors received any financial benefit for the conduct of the study. The Management of the University of Ibadan had no role in study design, data collection and analysis, decision to publish, or preparation of the manuscript. |
| 4. In your Data Availability statement, you have not specified where the minimal data set underlying the results described in your manuscript can be found. PLOS defines a study's minimal data set as the underlying data used to reach the conclusions drawn in the manuscript and any additional data required to replicate the reported study findings in their entirety. All PLOS journals require that the minimal data set be made fully available. For more information about our data policy, please see <http://journals.plos.org/plosone/s/data-availability>.   Upon re-submitting your revised manuscript, please upload your study’s minimal underlying data set as either Supporting Information files or to a stable, public repository and include the relevant URLs, DOIs, or accession numbers within your revised cover letter. For a list of acceptable repositories, please see http://journals.plos.org/plosone/s/data-availability#loc-recommended-repositories. Any potentially identifying patient information must be fully anonymized.  Important: If there are ethical or legal restrictions to sharing your data publicly, please explain these restrictions in detail. Please see our guidelines for more information on what we consider unacceptable restrictions to publicly sharing data: http://journals.plos.org/plosone/s/data-availability#loc-unacceptable-data-access-restrictions. Note that it is not acceptable for the authors to be the sole named individuals responsible for ensuring data access.  We will update your Data Availability statement to reflect the information you provide in your cover letter. | Thank you for the guidance. The study is a retrospective review of medical records only. The data analysed in the study is from a third party, the University Health Services (UHS), University of Ibadan and usage was approved by the Social Sciences and Humanities Research Ethics Committee (SSHREC), University of Ibadan, Nigeria [UI/SSHEC/2020/0021]. Data cannot be shared publicly because of the ethical and legal restrictions involving hospital records and patient information.  However, data may be made available with the permission of UHS management through the Director (contact: [dir_uhs@mail1.ui.edu.ng](mailto:dir_uhs@mail1.ui.edu.ng), ronkeajav@yahoo.com) following a reasonable request by researchers who meet the criteria for access to confidential data after appropriate protocol submission to SSHREC (contact via [as.jegede@mail.ui.edu.ng](mailto:as.jegede@mail.ui.edu.ng.), [sayjegede@gmail.com](mailto:sayjegede@gmail.com), referring to the UI/SSHEC/2020/0021). |
| 5. We note that you have stated that you will provide repository information for your data at acceptance. Should your manuscript be accepted for publication, we will hold it until you provide the relevant accession numbers or DOIs necessary to access your data. If you wish to make changes to your Data Availability statement, please describe these changes in your cover letter and we will update your Data Availability statement to reflect the information you provide. | We would appreciate it if this information could be modified to reflect the changes in our updated Data Availability statement above. Thank you. |
| 6. Please include your tables as part of your main manuscript and remove the individual files. Please note that supplementary tables (should remain/ be uploaded) as separate "supporting information" files. | This has been done. Thank you. |
|  |  |
| **Reviewers’ comments:** |  |
| 1. Is the manuscript technically sound, and do the data support the conclusions?  The manuscript must describe a technically sound piece of scientific research with data that supports the conclusions. Experiments must have been conducted rigorously, with appropriate controls, replication, and sample sizes. The conclusions must be drawn appropriately based on the data presented.   Reviewer #1: Yes  Reviewer #2: Yes | Thank you.  Thank you. |
| 2. Has the statistical analysis been performed appropriately and rigorously?   Reviewer #1: Yes  Reviewer #2: Yes | Thank you.  Thank you. |
| 3. Have the authors made all data underlying the findings in their manuscript fully available?  The [PLOS Data policy](http://www.plosone.org/static/policies.action#sharing) requires authors to make all data underlying the findings described in their manuscript fully available without restriction, with rare exception (please refer to the Data Availability Statement in the manuscript PDF file). The data should be provided as part of the manuscript or its supporting information or deposited to a public repository. For example, in addition to summary statistics, the data points behind means, medians and variance measures should be available. If there are restrictions on publicly sharing data—e.g. participant privacy or use of data from a third party—those must be specified.   Reviewer #1: Yes  Reviewer #2: No | The tables were uploaded separately and were erroneously omitted in the old manuscript PDF file. This has been updated and the tables have been included. |
| 4. Is the manuscript presented in an intelligible fashion and written in standard English?  PLOS ONE does not copyedit accepted manuscripts, so the language in submitted articles must be clear, correct, and unambiguous. Any typographical or grammatical errors should be corrected at revision, so please note any specific errors here.   Reviewer #1: Yes  Reviewer #2: No | The manuscript has been revised and all the typographical and grammatical errors have been corrected. Thank you. |
| **Reviewers' comments:** Reviewer #1: |  |
| 1. This is a very nice article analyzing the prevalence of obesity and its associated medical conditions in an African University. 2. The article states increased rates of obesity as a concern in LMIC in the starting introductory lines, however later charts/tables show a decreasing or stable trend and in fact a rising trend for underweight status. I agree that there is a double epidemic of under and over nutrition. However, this cannot be generalized to the population based on the prevalence numbers of this study which is only from university students. In the general population there may be a different trend which this study is not able to predict. This needs to be mentioned in the discussions as a limitation. 3. I do not think we can generalize the prevalence of an obesity epidemic from the study to the general population in the African subcontinent. I think this needs to be clearly mentioned as a limitation. Additionally, some of the sentences in the introduction seem to suggest an obesogenic environment in the University, however I am unsure how the authors drew such a conclusion with the data from the study showing an increase in underweight students in the enrollment category. Also, this article did not study a difference between the same students increasing in weight between their first and subsequent years of enrollment and hence is unable to draw on such a conclusion. I think the tone of the introduction could be limited to only mentioning the lack of prevalence data such as obesity, hypertension in male/female students and trends and the need to study this further. 4. It is unclear if the higher female enrollment is causing a rising trend in obesity, though less likely this needs to be noted in the discussions as a suggestion for future study and the reasons why? Is this because of pregnancy, marital status improved socioeconomic status? | Thank you for your kind words.  We found a consistently increased trend of overweight and obesity with age (table 4) with statistical significance. Line 302, 313-315. Also, there was an increasing shift from underweight to overnutrition (overweight/obesity) across each year of entry into the University (table 2, fig 3). Line 234. This information (fig 3) was inadvertently omitted initially but has been included in the revised manuscript as guided by your comment. Thank you  We agree that this study described a single-centre experience which may not reflect the same situation with the general population in the Africa continent, this limitation has been included in the revised manuscript. Line 567. However, our results fall in line with other findings about increasing high BMI status in adolescents and young adults in Nigeria and other developing countries in Africa.  The data in the study depicts the profile of a large population of adolescents, young and older adults who came in as fresh students from secondary schools and postgraduate students from the University of Ibadan (UI) and other tertiary institutions from different states and regions of Nigeria. The postgraduate students from UI were former undergraduates in UI who had returned for postgraduate studies and had their medical screening repeated during the period in review. UI, as the premier university in Nigeria, has a unique status and recruitment policy that covers and offers admission to eligible students from all the states and regions of Nigeria. Hence the data reflects a nationally diverse population of young persons in the country. In fact, the PAN-African University (PAU) which is hosted by UI consist of only postgraduate students from African countries, but, unfortunately, we couldn’t determine the distribution of these categories of postgraduate students (from UI, other tertiary institutions in Nigeria and those from PAU) because the data were totally anonymized before we accessed them. We have tried to update this information in our discussion. Line 157-165.  Regarding the comment on the obesogenic environment in the university, it is true we did not study the difference in the same student between the entry point and at any other time before graduating perse. However, the significant association between overweight/obesity prevalence and increasing age and postgraduate study gives room for some cautious inferences. First, some of the study participants who enrolled for postgraduate study were returning undergraduate students of UI who had their medical screening repeated for postgraduate admission during the period under review. Like many institutions, UI gives admission preference to their young students who have good academic records for postgraduate studies before considering students from other institutions. Unfortunately, we couldn’t synthesize this information in our data because of ethical restrictions (anonymized data). Whatever it was, this category of students may have been impacted by the UI school environment. Second, the result of some studies to date shows that there has not been any form of intervention for adolescents and young adults with high BMI in universities and the general population in Nigeria and other countries in Africa. Any institution where there is no decisive policy and interventions to tackle obesity could be defined as obesogenic. Therefore, both the undergraduate and postgraduate students admitted into UI and from tertiary institutions outside of UI who were previously underweight or overweight may naturally follow the life course of the obesity trajectory, all things being equal. This is one plausible way to explain the consistent and significant rise of overweight/obesity across the age groups and extending to the postgraduate years. Of course, this and others need further study as had been indicated in the discussion. Line 394-412.  In the study, we found an increasing trend of abnormal BMI (underweight, overweight, obesity) in females. Many reasons have been advanced in literature for this gender peculiarity, especially factors bothering on marital status, socio-cultural practices, economic disparity and those promoting gender inequality. However, our data do not contain information about any of these factors and are not part of the objectives of the study. We agree that this result and the likely causes need further examination. This point has been added to the discussion. Line 522-524. |
| 5. LVH may not be detected in early hypertension. It is a poor metric to determine presence of hypertension or the need for treatment. I think using the JNC criteria probably is a better way of assessing presence of hypertension and need to treat it. Given that this is a study of prevalence, I would suggest not mentioning left ventricular hypertrophy as this study was not designed to identify the prevalence of this as not all patients had an ECG. | Your comment about LVH is helpful. It has made us revise the manuscript more thoroughly to bring out the relevance of LVH to our findings beyond its prevalence. LVH is one of the earliest complications of sustained hypertension in children, young and older adults. Though present, it may not be detected because of under-assessment, inappropriate tools and poor health-seeking behaviour including late presentation – all or some of these are not far-fetched in our study. LVH (both eccentric and concentric types) is also a complication of overweight/obesity with or without hypertension. It is one of the associated chronic disease risks that our study elucidated. It is an independent risk factor for cardiovascular disease and a cause of sudden cardiac death in overweight and obese adolescent and young adult or athletes. In recent times, there has been an increase in the prevalence of sudden death among young people in Nigeria necessitating a call for mandatory medical screening and ECG for young people going for the mandatory 1-year National Youth Service Corps (NYSC) and preparticipation exercise programmes. Hence, LVH deserves special attention and discussion in our study. As it turned out in our study, obesity-associated chronic health risks including LVH were grossly under-assessed in the participants. Interestingly, LVH is an eminently modifiable risk factor for CVD. Line 467-492, 519-520.  We agree with your point that using JNC is adequate to assess the presence of hypertension and the need to treat it. Thank you. |
|  |  |
| **Reviewers' comments:** Reviewer #2: |  |
| 1. Dear author, while i congratulate you on the time and effort you have put into preparing this manuscript, I feel that there are significant changes that will improve the quality of your manuscript.   You will find below, detailed suggestions that you may find useful and decide to adopt.  I feel that these suggestions will improve the quality of your manuscript.  I will also advice that you make your work more concise so that the relevant information can be easily identified.  Your manuscript will benefit from the services of an English language editor. | Thank you for your kind words and detailed comments with suggestions. The manuscript is revised accordingly to improve the scientific writing and the quality of the manuscript.  Thank you for your comments and your helpful suggestions. The engagement and conciseness of the manuscript have been revised accordingly to reflect the suggested improvements. Overall, your comments and suggestions have enhanced our engagement, conciseness and the quality of our manuscript. |
| 2. Title: The title appears appropriate and descriptive of the study done. However, uniformity is required in the use of lower or upper case for letters which begin each word. It appears rather haphazard at the moment. Also, please write “10” as “Ten” | Apologies for the typos and grammatical errors. These have been corrected to read:  ‘’Temporal Trends in Obesity and Chronic disease risks Among Adolescents and Young Adults: A Ten-year Review at a Tertiary institution in Nigeria’’  Thank you |
| Abstract:   3. The abstract summarizes the work done and relevant outcome. A large majority was ≤ 40 years (95.1%) Line 53: should be change to “ Majority of the students samples were ……” 4. Line 58: Furthermore, females had a higher burden of coexisting abnormal BMI characterised by the co occurrence of underweight, overweight and obesity”.   Please give the exact values or the three abnormal BMI parameters in women.   5. Line 62: Hypertension is significantly associated with age, male sex, overweight/obesity and family history of hypertension. Was any test of statistical significance done? Please state this if any was done.   Did you mean increasing age?   6. Key words: seem to be appropriate | Thank you for this comment. The correction has been effected to read:  ‘The majority were ≤ 40 years (95.1%)’. Line 52.  Furthermore, females had a higher burden of coexisting abnormal BMI characterised by underweight (11.7%), overweight (20.2%) and obesity (10.4%). Line 56-58  Yes, statistical significance was done (p < 0.001). Line 61. Please see table 6  Yes, hypertension was significantly associated with older age, male sex, overweight/obesity and family history of hypertension (p = 0.001). Line 60-61  Thank you. The keywords have been updated to include ‘’Adolescents, Young adults’’ in the title, abstract and the body of the manuscript as you have rightly suggested below. |
| Introduction   7. Lines 92 and 93 and in other parts of the manuscript: Replace y” Young people “with the young”   The causes of excess weight gain in young people are similar to those in adults. However, young 93 people   8. Line 95: “ during leaving home.” Replace the “ at time of leaving home for university or college education. 9. I find the introduction rather too lengthy. It could be made more concise and much more to the point.   Study setting   10. Line 153: please delete “the major” 11. Line 179 : please change to “during the period in review” 12. Line 182 : please change “for each year of entry during the review 183 period.” to “…yearly for the period in review’ 13. Line 189: Under weight should be included as a variable. 14. Use the abbreviation UI to represent University of Ibadan after you have used the first abbreviation 15. Line 216: what is meant by “programme”? 16. Line 217: Consider deleting “large” . Simply say “ a majority” 17. Line 218: “a quarter of what’? Also, delete “the years under review”…this phrase has been used too often. 18. Lines 218 to 220 : Please reconstruct this sentence to make the meaning clearer. “In addition, the 219 percentage in this age group has been on the increasing trend since 2013 till 2018 while the reverse 220 pattern was observed for age 36 years and above of which their percentage declined”. 19. Lines 233 to 234: is not well written and has been repeated. Please re write. 20. Line 260: Except “ in students” 21. Line 314: Use “UI” 22. Line 315: Your sample categorization does not only include young adults, but older adults too. 23. Lines 329 to 330: This trend persists 329 till later years of postgraduate programmes depicting increasing risk of obesity at middle age and 330 later in adulthood. ( This sentence needs to be re written to be make it clearer) 24. Line 338: “ without investment in the built environment for physical activity.” The meaning of this is not clear. Please clarify. 25. Line 347: Please what is meant by “increasing shift”? Does this mean that under nutrition was decreasing and over nutrition was increasing as the year of entry increased? Please make your sentences clearer and easier to follow and understand. You can cite any of the figures that illustrates this and give a reason for this finding. 26. Line 343: The discussion in the section on Double Burden of Malnutrition should reflect the findings and numbers reported in the results. This is not the case presently. I will suggest a re-write of this section discussing the figures and relevance of both underweight and overweight, comparison with other reports, causes and implications to the wider society. 27. 403 : change tiny to the exact percentage> 28. 433 : “Young people” should be changed to “ Adolescents and young adults” all over the manuscript as has been used previously in your manuscript. 29. Though this manuscript’s title focuses on Obesity, much more ought to have been said about under weight (under nutrition ) 30. Interventions : This will include education of the harmful effect of poor nutrition and advice and on the benefits of healthy eating including components of a balanced diet. The positive effect of role models. Deterrents for higher energy drinks and policy statements such as increased tax on such high energy drinks etc 31. Though i could find the figures, there were no tables present for inspection. 32. A major flaw of this manuscript is that the author’s posture and discussion is focused on the students and their learning environment (UI). This manuscript gives the impression or idea that these are students who have been in UI. However, this is not the case. The sample used are fresh students who have been out of the school and are just resuming into UI. Therefore, this sample is not affected or do not have any impact yet by the school environment. The BMI measurements were taken at the point of admission into the school and not during their study. Therefore, this does not reflect the impact of UI environment as the authors have made it appear. This should be corrected all over the manuscript. 33. The other issue is that the manuscript is much too long and almost boring. The information can be given in fewer words and in clear sentences too. The authors should engage an English language editor to help make their work clearer and more focused. 34. I will also suggest that the tables be included in the submission. | This has been done. Line 96 and 97. Thank you.  The causes of excess weight gain in the young are similar to those in adults. However, the young are significantly prone to obesity…Line 97  …lifestyle changes that occur at the time of leaving home for university or college education. Line 99  The introduction has been reduced from five to four paragraphs and rephrased concisely.  ‘’the major’’ deleted to read ‘from the centre of Ibadan city in Southwestern Nigeria.’ Line 144  medical records during the period in review was collected. Line 156.  …and 3,000 postgraduate students yearly during the period in review. Line 165  The outcome variables of interest were underweight, overweight and…Line 190  This has been done. Thank you  This is about the programme mode (whether undergraduate or postgraduate). Line 215.  The majority of the students were ≤ 40 years (95.1%). Line 217.  Apologies for this incomplete statement, we have rephrased the statement as ‘’ Students aged 16-20 years constituted at least a quarter of the study population. The phrase ‘’the years under review’’ also deleted accordingly. Line 217.  In addition, the percentage of participants aged 16-20 years increased steadily from 34.9% in 2013 to 53.8% in 2018, while those aged 36-40 years declined from 4.1% to 1.9%. Line 218-219.  Thank you for pointing out this error due to the insertion of another sentence. This has been corrected as:  Similarly, the percentage of obese individuals ranged between 9.2% in 2012 and 5.0% in 2014. Line 233.  The sentence has been rephrased: ‘’students that were overweight or obese increased as age increased’’. The ‘’except in students’’ expunged. Line 303.  ..admitted into UI. Line 380.  Thank you for pointing out this important fact. The sentence has been revised to read ‘’a large population of adolescents, young and older adults’’. Line 379.  This sentence has been rephrased thus:  ‘’We found a consistently increasing trend of overweight and obesity with age, which persists significantly in many of the study participants till the later years of their postgraduate study, suggesting an increased risk of obesity till middle age and later in adulthood’’. Line 394-396.  We have addressed this unintended confusing statement by rephrasing the sentence as: ‘’with the lack of investment in developing an environment that promotes physical activity’’. Line 408.  No, undernutrition was not decreasing but rather, there was a rising trend in the underweight status of the participants as the year of entry increased. However, while undernutrition was increasing, there was an increasing shift from underweight to overnutrition (overweight/obesity) simultaneously across each year of admission to the University (table 2, figure 3). Line 416-418. The is the dual burden of malnutrition phenomenon due to nutrition and economic transitions in developing countries. Line 428-430. It’s regrettable that this information (fig 3) was inadvertently omitted initially but has been included in the revised manuscript as guided by your comment. Thank you  Thank you for your comments and suggestion that have stimulated further discussion on the double burden of malnutrition. We have included more discussions on the dual burden of malnutrition, the relationship and the biological link between the diverse forms of malnutrition beyond coexistence. However, underweight was the only index of undernutrition that was explored in this study. Other indices of undernutrition – stunting, wasting and micronutrient deficiencies were not part of the outcome variables for this study and may not be sufficiently supported by our data. We did not find a significant association between underweight and chronic disease risks. However, we presented the possible complications and public health implications of undernutrition and the need for more investigations in future studies. Line 434-453.  This study shows that only 5.6% of the study participants had Electrocardiography. Line 471.  This comment is well noted and has been addressed where appropriate. Thank you.  We have included more discussions on underweight as stated in line 437-453.  These points are well appreciated and are part of our discussion. Line 524-528, 544-547.  We regret the error that occurred, the tables have been included in the revised manuscript.  Thank you for this comment. It reflects how much more we need to do to communicate some peculiar aspects of the study population and environment. First, some of the postgraduate students were former UI undergraduates who had returned for postgraduate studies and had their medical screening repeated. Like many institutions, UI gives admission preference to their young students with good academic records for postgraduate studies before considering students from other institutions. Whatever it was, this category of students may have been impacted by the UI school environment. Second, the remaining postgraduate students too were not just coming from secondary school but other Nigerian tertiary institutions. Wherever they came from, the impact of their school environment on this other category of postgraduate students cannot be ignored because it couldn’t have been zero. Overall, the postgraduate students consisted of 48.1% of the study population.  Regarding the comment on the potential obesogenic environment in UI, the significant association between overweight/obesity prevalence and older age and postgraduate study gives room for some cautious inferences. First, it bears repeating that some of the study participants who enrolled for postgraduate study were returning undergraduate students of UI who had their medical screening repeated for postgraduate admission during the period under review. Unfortunately, we couldn’t synthesize this information from our study because the data was fully anonymized. Second, the result of some studies to date show that there has not been any form of intervention for adolescents and young adults with high BMI in universities and the general population in Nigeria and Africa. Any institution where there is no decisive policy and interventions to tackle obesity could be defined as obesogenic. Moreover, studies show that university students lack knowledge about healthy food choices. Therefore, both undergraduate students and postgraduate students admitted through UI or from tertiary institutions other than UI who were previously underweight or overweight may naturally follow the life course of the obesity trajectory, all things being equal. This is one plausible way to explain the consistent and significant rise of overweight/obesity across the age groups and during the postgraduate years. Of course, this and others need further study.  Consequently, we have made another attempt at revising the manuscript to reflect the peculiar characteristics of the study environment and participants in our discussion. Line 157-165, 396-412. Thank you very much.  Thank you. This point has been addressed in your previous comment on the introduction section and also in the body of the manuscript.    This point is well noted and has been addressed in the revised manuscript. Thank you. |
