# Supplementary material for: Temporal trends in overweight and obesity and chronic disease risks among adolescents and young adults: A ten-year review at a tertiary institution in Nigeria
Source: PLoS One. 2023 Apr 5;18(4):e0283210. doi: 10.1371/journal.pone.0283210 (PMC10075485; doi:10.1371/journal.pone.0283210)
Supplement: Attachment — Submitted filename: Response to Reviewers R2.docx [file pone.0283210.s002.docx]

November 29, 2022

**Rebuttal letter: response to reviewers**

PONE-D-22-08089R1

**Temporal Trends in Obesity and Chronic disease risks Among Adolescents and Young Adults: A Ten-year Review at a Tertiary Institution in Nigeria**

PLOS ONE

**Journal Requirements:**

Please review your reference list to ensure that it is complete and correct. If you have cited papers that have been retracted, please include the rationale for doing so in the manuscript text, or remove these references and replace them with relevant current references. Any changes to the reference list should be mentioned in the rebuttal letter that accompanies your revised manuscript. If you need to cite a retracted article, indicate the article’s retracted status in the References list and also include a citation and full reference for the retraction notice.

Author’s Response: Thank you for your review. The reference list has been revised and updated to reflect the changes in the manuscript text which was overhauled to make it more concise as variously suggested. The changes to the whole reference list are as tracked in the file labeled “Revised Manuscript with Tracked Changes”

**Review Comments to the Author**

Reviewer #1: The suggested revisions have been accepted or manuscript edited for those suggestions. Paper would benefit from being concise.

Author’s Response: Thank you for your painstaking review and practical suggestions so far. The manuscript has undergone further review to make it more concise and improve engagement. Some sentences and paragraphs were modified or totally expunged. The modifications and changes in the following areas are noteworthy:

**Introduction, line 68 in the revised manuscript**

The second paragraph of this latest revision ends in line 93 with the expunging of “This dual burden of malnutrition places heavy tolls on individuals, families, economies, and healthcare systems [22,23,24]” in lines 93 to 94 in the manuscript with tracked changes.

The third paragraph starting from line 95 was also reviewed and reduced by expunging (lines 102 to 105 in the manuscript with tracked changes), “For example, there is a preference for energy-dense foods, higher-fat intake, and more sugar-containing drinks. Reduced energy expenditure due to insufficient physical activity and sedentary activities such as long hours of watching television or other screen devices are also contributory factors in an increasingly urbanised and digitalized world.”

The fourth paragraph begins in line 106 with “The period of stay…”. Lines 111 to 112 are expunged in the manuscript with tracked changes. Also expunged is a statement in line 122, “It may as well be that universities are unwittingly sustaining an obesogenic environment critical to amplifying the biological vulnerability of young people to obesity.”

**Discussion**

***The burden of overweight and obesity, line 377 in the revised manuscript***

The second paragraph, line 395 in the manuscript with tracked changes was modified and rearranged. The first sentence was modified as, “We found a consistently increasing trend of overweight and obesity with age, which persists significantly in the consecutive admission cohorts, suggesting an increased……” This was followed by a rearrangement of the paragraph after expunging a large chunk of many sentences/statements in lines 408 to 415 in the tracked manuscript.

***Double Burden of Malnutrition, line 400 in the revised manuscript***

The first and second paragraphs in this sub-heading were reviewed and redundant statements were removed, lines 428 to 433 in the first paragraph and lines 435 to 437 and 441 to 443 in the manuscript with tracked changes.

***Obesity-related health conditions, line 430 in the revised manuscript***

The second paragraph in this sub-heading starting in line 441 (472 in the manuscript with tracked changes) was rearranged/re-paraphrased with the removal of some statements.

Reviewer #2: Dear Authors, I congratulate you on the efforts you have put into this manuscript. It is improved. I only have the following observations:

1. The introduction can still be made even more concise.

Author’s Response: Thank you so much for your review, suggestions, and kind words. The introduction and some other sections of the manuscript have been revised, modified, re-paraphrased and some statements expunged as follows:

**Introduction, line 68 in the revised manuscript**

The second paragraph of this latest revision ends in line 93 with the expunging of “This dual burden of malnutrition places heavy tolls on individuals, families, economies, and healthcare systems [22,23,24]” in lines 93 to 94 in the manuscript with tracked changes.

The third paragraph starting from line 95 was also reviewed and reduced by expunging (lines 102 to 105 in the manuscript with tracked changes), “For example, there is a preference for energy-dense foods, higher-fat intake, and more sugar-containing drinks. Reduced energy expenditure due to insufficient physical activity and sedentary activities such as long hours of watching television or other screen devices are also contributory factors in an increasingly urbanised and digitalized world.”

The fourth paragraph begins in line 106 with “The period of stay…”. Lines 111 to 112 are expunged in the manuscript with tracked changes. Also expunged is a statement in line 122, “It may as well be that universities are unwittingly sustaining an obesogenic environment critical to amplifying the biological vulnerability of young people to obesity.”

**Discussion**

***The burden of overweight and obesity, line 377 in the revised manuscript***

The second paragraph, line 395 in the manuscript with tracked changes was modified and rearranged. The first sentence was modified as, “We found a consistently increasing trend of overweight and obesity with age, which persists significantly in the consecutive admission cohorts, suggesting an increased……” This was followed by a rearrangement of the paragraph after expunging a large chunk of many sentences/statements in lines 408 to 415 in the tracked manuscript.

***Double Burden of Malnutrition, line 400 in the revised manuscript***

The first and second paragraphs in this sub-heading were reviewed and redundant statements were removed, lines 428 to 433 in the first paragraph and lines 435 to 437 and 441 to 443 in the manuscript with tracked changes.

***Obesity-related health conditions, line 430 in the revised manuscript***

The second paragraph in this sub-heading starting in line 441 (472 in the manuscript with tracked changes) was rearranged/re-paraphrased with the removal of some statements.

2. Line 150: The University commenced in 2011….. ( Which University is referred to here, UI or PAU?)

Author’s response: The statement here refers to PAU and this has been corrected in line 142 in the revised manuscript.

Thank you.

3. Line 230: …….between 2009 and 2017 (Based on Figure 1, there was a decrease in 2018 compared to the 2016 and 2017. I therefore recommend stating between 2009 and 2017)

Author’s Response: This correction has been done. Line 222 in the revised manuscript.

Thank you.

**Other significant modifications**

Line 218 in the manuscript with tracked changes – Students aged 16-20 years constituted at least a quarter of the study population. This statement was changed to: Students aged 16-20 years constituted over a third of the study population, line 209 in the revised manuscript.

Line 567 in the manuscript with tracked changes – Furthermore, the study spans a ten-year period which shows trends in obesity and hypertension, thus increasing the validity and power of this study. This statement was changed to: Furthermore, the study spans a ten-year period showing the trajectory of obesity and hypertension prevalence.

Thank you
