# Supplementary material for: Temporal trends in overweight and obesity and chronic disease risks among adolescents and young adults: A ten-year review at a tertiary institution in Nigeria
Source: PLoS One. 2023 Apr 5;18(4):e0283210. doi: 10.1371/journal.pone.0283210 (PMC10075485; doi:10.1371/journal.pone.0283210)
Supplement: Attachment — Submitted filename: Response to Reviewers Feb 2023.docx [file pone.0283210.s003.docx]

**Additional Editor Comments:**

In data analysis, the author(s) mentioned, “Trends in obesity and hypertension were assessed using Chi-square for trends at a 5% significance level. Independent factors associated with obesity/overweight and hypertension were evaluated using a multivariable binary logit model. Measures of effect were reported as Odds Ratio with 95% Confidence Interval (95% CI)”. I suggest rewriting it all. Chi-square is used to measure association, not trends. In the second line, if the factors are independent then why are you finding the association? Change word “independent”. Odds ratios are also used for finding the association. Measure of effect is not a suitable world.

Response. Thank you for your observation and suggestions.

Line 197, independent removed

Line 198, effect removed, association inserted.

The section has been revised accordingly to read:

The prevalence of overweight and obesity and the trends during the period in review were determined. The association between the students’ sociodemographic characteristics, hypertension and overweight/obesity was assessed using Chi-square at a 5% significance level. The factors associated with obesity/overweight and hypertension were evaluated using a multivariable binary logit model. Measures of association were reported as Odds Ratio with a 95% Confidence Interval (95% CI).

Editor. Table 1 provided (%) but table 2 provided n(%). I suggest presenting both.

Response: n(%) was provided in table 1, line 233

Line 238, fig 1 cancelled, table 2 inserted

Editor. Table 4 presents chi-square, but the proper notation of chi instead of writing X

Response: Chi-square proper notation inserted in line 197 and used in Tables 4 and 6. Thank you.

Editor. “Table 5: Multivariate analysis showing the association between socio-demographic 312 characteristics of students and Overweight and Obesity” should be, “… of students with
I am wondering to see that all p-values in tables 5 and 7 are exactly 0.001.

Response: Those were the exact p-values obtained. This is not unusual given the very large sample size that we had.

The title for table 5 has been revised, ‘with’ substituted for ‘and’ line 312

Editor. Table 6, for pre-hypertension, there is only a yes category in the header.

Response. A ‘no’ category was added, line 327. Thank you.

Editor. Table 7 has a different font size.

Response. The font size was increased and aligned with other tables.

Editor. Figure 3 has a title “chart title”

Response. The “chart title” was removed and figure 3 converted to figure 1.

Editor. Figure 5 has a dotted trend line, which is not appropriate for bar graphs and each of the year categories has the repeated word “year.”

Response. Figure 5 was revised accordingly but completely removed because it didn’t provide any additional information or value more than what the tables provided. Thank you.

Editor. Figure 1 and figure 2 are both combined in figure 3 so I think no need for figures 1 and 2.

This is very true, figures 1 and 2 were removed. Thank you.

Editor. In Figures 4 and 2, the author(s) presents the results for underweight, overweight, and obese. But the table only compares overweight and obese as yes or no. Why did the author(s) not measure the association with underweight, normal, overweight, and obese instead of using combined overweight as yes/no? Author(s) discussed the overweight and obese together, although the paper title has the obese only. While WHO highly encourages to deal overweight and obese as different categories.

Response. Undernutrition and its associations have historically received more public health attention than over-nutrition in sub-Saharan Africa, especially in Nigeria. Also, there are intervention studies and programmes on undernutrition, including the ongoing national school feeding programme by the government of Nigeria. However, overnutrition is a growing, silent epidemic among children and young people with scarce data and without attention and intervention yet. Besides, our study is not just about the prevalence of overweight or obesity but its associations with chronic disease risks. The data for our study is restrictive as it lacks adiposity measures beyond BMI and cannot support robust research analysis to generate (new) findings on the various forms and manifestations of undernutrition and chronic disease risk associations. Hence, the objectives of our study focused mainly on overnutrition based on BMI (the only available indicator of adiposity), and we did not intend to repeat research on undernutrition or seek associations between underweight and chronic disease risks which could not be supported by our data. Even for the normal category, without additional adiposity measures for proper categorization, it may yield misleading results. These were the reasons why we did not include underweight and normal in the table. Figures 4 and 2 were used suitably for the purpose of comparison only.

Combined category

First, the combined category of “overweight and obese” refers to a BMI of 25 or higher. Overweight and obesity are defined as abnormal or excessive fat accumulation that may impair health. The combined category is preferably used to avoid underestimation of measures when the value (absolute number or percentage) of the separate categories is low as we have in our data. Compared with the overall prevalence, there was a marked decrease in the absolute numbers and percentages of the separate categories of overweight and obese, corresponding with the lower postgraduate admission rate during some years in the period in review. The obese category is most significantly reduced with the lowest rate of postgraduate admission, especially in 2017. Second, this combination is also expedient in our study since there are no additional measures of adiposity (information on fat location/distribution) that can be used to estimate the risk for associated NCDs except for high BMI. The key difference between being overweight and obese lies in the extent of accumulation of excessive body fats and this does not give information about its location/distribution. Except for those with an exceeding large total fat mass (BMI), where fat is accumulated/distributed is much more important than the extent (amount) of the separate BMI categories in the prediction of comorbidities such as cardiovascular disease, diabetes, hypertension, malignancies, or overall death rates. This is one of the well-documented limitations of BMI as an indicator of body fat and associated chronic disease risk.

Obesity, defined as a disease or a medical condition that poses risk to health, is a generic term that includes overweight and obese and/or other categories of obesity. Obese or overweight are separate categories of obesity defined by BMI only (anthropometric obesity). Also, Obesity is categorised not only by BMI but other measures like waist circumference, waist-hip ratio (central or abdominal obesity). Obesity includes individuals with normal BMI but are obese based on these other measures. So, the term obesity in our title is not restricted to obese only but covers both overweight and obese. However, based on your comment, we added overweight to the title of our study, to read and be interpreted as, the temporal trends in overweight (as a distinct/separate BMI category) and obesity (as a combined BMI category of overweight and obese)…. Thank you.

**Reviewer #2:**

Dear Author, your work is much improved.
However, i feel that in line with the title of your paper, suggesting that the trends are being examined, table 2 clearly suggests an increase in underweight ( the absolute numbers and percentages of underweight for the first five years of the study period are less than for the later five years ) and a decrease in both overweight and obesity (the absolute numbers and percentages of both over weight and obesity for the first five years are more than for the last five years). This ought to be clearly mentioned and discussed.
Also this appears to contrary to the statement in line 226 and 227 "On the other hand, there was an increasing shift from underweight to overnutrition (overweight and obesity) across each year of admission into the University.

Response. Truly, when compared with the overall percentage and number, there is a marked decrease in the absolute numbers and percentages of both overweight and obese in 2014, 2016 and 2017 (now included in line 242) corresponding with the lower postgraduate admission rate during these years. Despite the obvious dip in the values of overweight and obesity during these years, the prevalence of overweight was consistently higher than underweight, except for 2017 (Table 2, line 243). The validity of the statement about the transition from undernutrition to overnutrition across each admission year is sustainable (fig 1). It is difficult and beyond the scope of our study to adduce any reasons for the change in either the admission or medical registration pattern or discuss its implications differently. However, “an increasing” shift from underweight to overnutrition was changed to a shift from underweight to overnutrition (line 244, 407).

Thank you for all your comments and valuable suggestions.

Reviewer #2. Though cancer is mentioned as one of the variables sought after in the records in line 185, this is not reported on in the results. Please state the number of cancer diagnosed and if none then mention it.

Response. There was no record of cancer diagnosis. This statement has been added in line 266.

Thank you.

**General corrections:**

Title: overweight was added as explained in the Editor’s comment above.

Abstract

line 58 obesity (10.4%) changed to obese (10.4%)

line 63 ‘rising trends in prevalence’ was canceled and inserted, a higher prevalence of overweight and obesity than underweight among the participants.

Thank you.
